# The tumor suppressor RhoBTB1 controls Golgi integrity and breast cancer cell invasion through METTL7B

**DOI:** 10.1186/s12885-017-3138-3

**Published:** 2017-02-20

**Authors:** Caroline M. McKinnon, Harry Mellor

**Affiliations:** School of Biochemistry, Biomedical Sciences Building, University Walk, University of Bristol, Bristol, UK

**Keywords:** Rho GTPases, RhoBTB1, BTB domain, Methyltransferase, Golgi fragmentation, Cell migration, Cell invasion

## Abstract

**Background:**

RhoBTB1 and 2 are atypical members of the Rho GTPase family of signaling proteins. Unlike other Rho GTPases, RhoBTB1 and 2 undergo silencing or mutation in a wide range of epithelial cancers; however, little is known about the consequences of this loss of function.

**Methods:**

We analyzed transcriptome data to identify transcriptional targets of RhoBTB2. We verified these using Q-PCR and then used gene silencing and cell imaging to determine the cellular function of these targets downstream of RhoBTB signaling.

**Results:**

RhoBTB1 and 2 regulate the expression of the methyltransferases METTL7B and METTL7A, respectively. RhoBTB1 regulates the integrity of the Golgi complex through METTL7B. RhoBTB1 is required for expression of METTL7B and silencing of either protein leads to fragmentation of the Golgi. Loss of RhoBTB1 expression is linked to Golgi fragmentation in breast cancer cells. Restoration of normal RhoBTB1 expression rescues Golgi morphology and dramatically inhibits breast cancer cell invasion.

**Conclusion:**

Loss of RhoBTB1 expression in breast cancer cells leads to Golgi fragmentation and hence loss of normal polarity.

**Electronic supplementary material:**

The online version of this article (doi:10.1186/s12885-017-3138-3) contains supplementary material, which is available to authorized users.

## Background

The Rho GTPase family of signaling proteins are master regulators of cell shape and cell migration. They do this directly through dynamic regulation of the actin cytoskeleton; however, they also have diverse additional cellular roles that contribute to this, including the control of membrane trafficking, cell polarity and gene expression [1]. The roles of Rho GTPases in cell migration make them important signaling proteins in cancer. While Rho GTPases are generally not direct targets of mutation in cancer, their signaling pathways are frequently deregulated, promoting the switch to cancer cell invasion and metastasis [2, 3].

The human Rho GTPase family contains 20 members, of which RhoA, Rac1 and Cdc42 are the best characterized [4]. These are small, globular proteins whose activity is controlled by binding of GTP, which switches them into their active conformation. The Rho family also contains two atypical members – RhoBTB1 and 2. These are larger, multimodular Rho GTPases that have a conserved N-terminal Rho GTPase domain, but also two copies of the BTB (Broad-Complex, Tramtrack and Bric a brac) domain and a carboxyl terminal BACK (BTB and C-terminal Kelch) domain [5, 6]. Intriguingly, both genes undergo silencing or mutation in human cancer. Hamaguchi and colleagues identified RhoBTB2 in a representational difference analysis screen for novel tumor suppressor genes in breast cancer, and gave it the alternative name DBC2 (deleted in breast cancer 2). The RhoBTB2/DBC2 gene undergoes homologous deletion in a relatively small number of breast tumor samples; however, RhoBTB2 expression is silenced at high frequency (approximately 50%) in breast and lung tumors [7]. Subsequent studies have reported the silencing of RhoBTB2 expression in a wide range of human tumors, as well as sporadic point mutations of the RhoBTB2 coding region and promoter [8–11]. RhoBTB1 is 73% identical to RhoBTB2 at the protein level. Far less is known about its cellular functions; however, recent studies have shown that it is also downregulated in human cancers. It is subject to loss of heterozygosity at high frequency in head and neck squamous cell (HNSC) carcinomas [12] and its expression is silenced in colon cancer through the actions of the microRNA miR-31 [13].

Unlike the majority of members of the Rho GTPase family, RhoBTB1 and 2 do not regulate the actin cytoskeleton directly [14]. Many proteins with BTB domains function as transcription regulators [15] and in our previous studies we showed that this is also the case for RhoBTB2 [16]. To determine transcription targets of RhoBTB2, we silenced its expression in primary lung epithelial cells and then performed whole-genome microarray analysis of gene expression. This allowed us to identify the chemokine CXCL14 as a target of RhoBTB2 regulation [16]. CXCL14 expression is downregulated in a high percentage of carcinomas, and especially in HNSC carcinomas where its loss is correlated with poor prognosis. Importantly, we found that loss of RhoBTB2 expression is correlated with loss of CXCL14 expression in HNSC cancer cell lines, and that expression of the chemokine is rescued by re-expression of RhoBTB2 [16].

CXCL14 was the most significant hit in the RhoBTB2 microarray screen; however, several other genes also showed reduced expression upon RhoBTB2 silencing. One of these was METTL7A, a poorly-characterized methyltransferase enzyme. In this study, we investigate the regulation of the METTL7 enzymes by RhoBTB proteins and uncover a pathway controlling Golgi integrity in mammary epithelial cells.

## Methods

### Materials

Full details of antibodies, oligonucleotides and plasmids used in this study are given in Additional file 1.

### Cell culture and transfection

HeLa, HEK293T, MDA-MB-231, MCF7 and T47D cells were cultured in DMEM containing 10% heat-inactivated fetal bovine serum. HMT-S1 and MCF10A cells were cultured as previously described [17, 18]. HeLa cells were transfected with plasmids and siRNA oligonucleotides using calcium phosphate [16].

### Real-time PCR

RNA was isolated from cells using the TRIzol extraction method (Invitrogen) and 40 μg of purified RNA used for reverse transcription using Omniscript RTase (Qiagen) for 1 h at 37 °C. cDNAs were then subjected to real-time PCR using DyNAmo Flash SYBR Green (Finnzymes). Amplification was performed using an Opticon 2 thermocycler (MJ Research) and data was analyzed using the comparative Ct method.

### Immunofluorescence microscopy

Cells were fixed for 15 min in 4% paraformaldehyde in PBS and then permeabilized in 0.2% Triton X-100 in PBS for 5 min. Cells were then incubated with 0.1% sodium borohydride for 10 min. Primary antibodies were incubated with cells in 1% BSA for 1 h followed by secondary antibodies for 45 min. The cells were stained with 2 μg/ml DAPI for 10 min and mounted over MOWIOL 4–88 (Calbiochem) containing 0.6% 1,4-diazabicyclo-(2.2.2) octane as an anti-photobleaching agent. Confocal microscopy was performed using a Leica TCS-NT confocal laser-scanning microscope with an attached Leica DMRBE upright epifluorescence microscope under a PlanApo x63/1.32 oil-immersion objective. A series of images were taken at 0.5 μm intervals through the Z-plane of the cells and processed to form a projected image.

### Analysis of Golgi fragmentation

Fragmentation of the Golgi ribbon was scored blind in cells stained for the Golgi marker giantin. Fifty cells were scored from each condition to give the percentage of cells with a fragmented Golgi. Data from three independent experiments were processed to give the mean.

### Re-expression of RhoBTB1

Expression of RhoBTB1 was restored in T47D cells by stable integration of a lentiviral RhoBTB1 construct. mCherry-tagged RhoBTB1 was subcloned into pHR’SIN-cPPT-SEW [19]. Virus was generated by transfection of HEK293T cells as described [19]. Briefly, 40 μg of RhoBTB1 vector was mixed with 10 μg of envelope plasmid pMDG, 30 μg of packaging plasmid psPAX2 and 1 μl of 1 mM polyethylenimine (Sigma) in OptiMEM (Invitrogen). This transfection mixture was replaced 4 h later with 15 ml of normal culture medium. The cells were then incubated for 48 h to allow virus production. After the incubation, the medium was removed and centrifuged for 10 min at 2,600 x *g*. The supernatant was then passed through a 0.45 μm filter and this filtrate was used as the virus stock. T47D cells were transduced with virus stock by overnight incubation.

### Cell migration assays

T47D cells were grown to confluence in chamber slides (Ibidi). The confluent monolayer was scratched with a sterile pipette tip and migration was followed by brightfield imaging at 37 °C for 14 h using a Leica AF6000 live cell imaging workstation and x40 objective. Multipoint revisiting allowed for parallel imaging of samples. Average cell velocity was calculated using ImageJ (NIH). For quantification of Golgi polarization, cells were fixed 1 h after making the scratch and stained for giantin. Cell polarization was assessed by dividing cells into quadrants centered on the nucleus. Cells were judged to have polarized if the majority of their Golgi apparatus resided within the quadrant facing the scratch edge.

### Cell invasion assays

Invasion of T47D cells was quantified using BD BioCoat Matrigel Invasion Chambers (BD Biosciences) according to the manufacturer’s protocol. Briefly, T47D cells in serum-free medium were plated at 1 × 10^5^ cells per chamber. Medium containing 10% fetal bovine serum was used as the chemoattractant in the lower chamber. After 24 h, non-invaded cells were removed from the upper chamber using a cotton swab. Cells that invaded through the Matrigel to the bottom of the insert were fixed and stained by incubation with Diff-Quick (BD Biosciences) for 10 min. These stained cells were washed with PBS, air-dried and counted.

### Statistical analysis

Analysis of statistical significance was performed using GraphPad Prism software. Comparison of two sample experiments was by paired, two-tailed Student’s *t*-test. Analysis of multiple samples was made using one-way ANOVA. For comparisons to a control, Dunnett’s post hoc test was used, whereas comparisons between all samples employed Tukey’s post hoc test.

## Results

### RhoBTBs control the expression of METTL7 isoforms

**Fig. 1:**
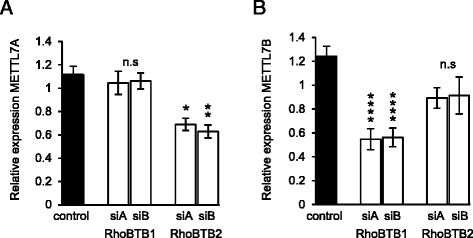
RhoBTBs differentially control METTL7 expression. **a** HeLa cells were transfected with siRNAs targeting RhoBTB1 or RhoBTB2, or the lamin siRNA control. Two independent siRNAs were used for each target. The efficiency of these siRNAs in HeLa cells was quantified in our previous study [16]. After 48 h, RNA was prepared from the cells and the expression of METTL7A was quantified by RT-PCR. Silencing of RhoBTB2 significantly reduced the expression of METTL7A. **b** Expression of METTL7B was quantified in the same samples. Silencing of RhoBTB1 significantly reduced the expression of METTL7B. Data are means ± SEM (*n* = 3). Data were normalized to mock-transfected HeLa cells. Comparisons are to the lamin siRNA control; *P <0.05, **P <0.01, ****P <0.0001

Our previous microarray screen identified METTL7A as a potential target of RhoBTB2-mediated transcriptional regulation [16]. To validate this, we used two independent siRNAs to silence RhoBTB2 in HeLa cells and measured METTL7A expression by RT-PCR. We also examined the expression of METTL7B, a closely-related protein that shares 59% sequence identity with METTL7A. Silencing of RhoBTB2 reduced the expression of METTL7A by approximately 30% (Fig. 1a). Previously, we showed that both RhoBTB1 and RhoBTB2 are required for expression of CXCL14 [16]; however, silencing of RhoBTB1 had no effect on METTLA expression (Fig. 1b). Interestingly, RhoBTB1 instead regulated the expression of METTL7B (Fig. 1b). We conclude that the two human METTL7 isoforms are differentially regulated by these two RhoBTBs.

### RhoBTB1 regulates Golgi integrity

**Fig. 2:**
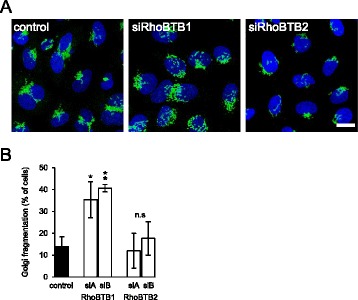
Silencing of RhoBTB1 causes Golgi fragmentation. HeLa cells were transfected with siRNAs targeting RhoBTB1 or RhoBTB2. After 48 h, the cells were fixed and stained for the Golgi marker giantin (*green*). **a** Shows representative images of normal and RhoBTB1 depleted cells. Bar = 10 μm. **b** Silencing of RhoBTB1 caused a marked and significant fragmentation of the Golgi. Data are means ± SEM (*n* = 3). Data were normalized to mock-transfected HeLa cells. Comparisons are to the lamin siRNA control; *P <0.05, **P <0.01

METTL7B was originally identified as a Golgi-associated methyltransferase of unknown function [20]. Previous work has shown that inhibition of cellular methylation leads to fragmentation of Golgi, although the identity of the methyltransferase involved is unknown [21]. We hypothesized that the RhoBTBs might act through METTL7 proteins to control some aspect of Golgi function. To examine this, we silenced RhoBTB1 and RhoBTB2 expression and examined the basic morphology of the Golgi by staining cells for the well-characterized Golgi marker, giantin. In control cells, the Golgi was present as a characteristic ribbon situated next to the nucleus. Silencing of RhoBTB2 had a negligible effect on Golgi morphology; however, silencing of RhoBTB1 caused profound fragmentation of the Golgi (Fig. 2a, b).

### RhoBTB1 regulates Golgi integrity through METTL7B

**Fig. 3:**
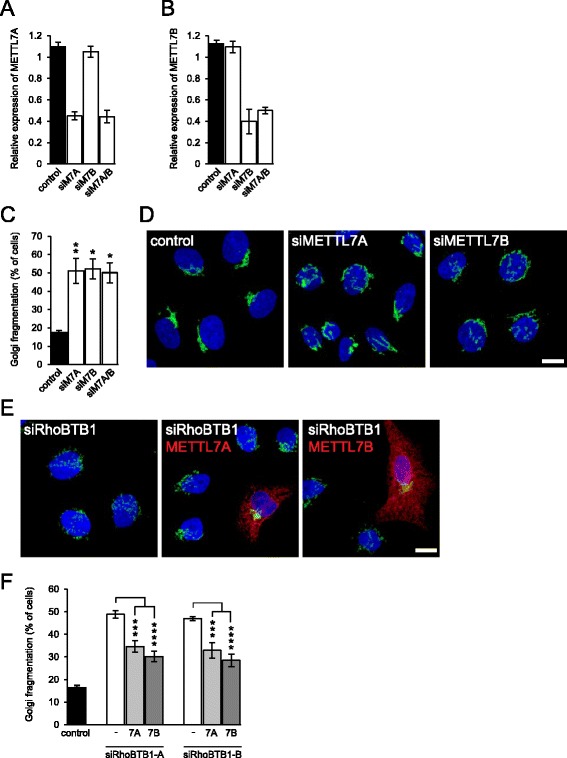
RhoBTB1 regulates Golgi integrity through METTL7 proteins. **a-b** HeLa cells were transfected with siRNAs targeting METTL7A, METTLB or both. After 48 h, RNA was prepared from the cells and the expression of each METTL7 was quantified by RT-PCR. Data are means ± SEM (*n* = 3). **c** HeLa cells were transfected with the same siRNAs and fixed and stained for the Golgi marker giantin. Silencing of either methyltransferase caused a marked and significant fragmentation of the Golgi. Data are means ± SEM (*n* = 3). Comparisons are to the control lamin siRNA. **d** Shows representative images of the cells. Golgi are stained in *green*; nuclei in *blue*. **e** HeLa cells were transfected with siRNAs targeting RhoBTB1 and with plasmids expressing myc-tagged METTL7A or METTL7B. After 48 h, the cells were fixed and stained for the Golgi marker giantin (*green*) and for the transfected methyltransferases (*red*). Bars = 10 μm. **f** Quantification of transfected cells showed that expression of either METTL7 rescued Golgi morphology in cells depleted of RhoBTB1. Data are means ± SEM (*n* = 3); comparisons are as indicated. *P <0.05; **P <0.01, ***P <0.001, ****P <0.0001

We designed specific siRNAs to each METTL7 isoform to allow us to investigate their role in the regulation of Golgi integrity (Fig. 3a, b). Interestingly, silencing of either isoform caused significant fragmentation of the Golgi (Fig. 3c, d). This raised the possibility that downregulation of METTL7B might underlie the effects of RhoBTB1 depletion on Golgi integrity. To test this, we silenced RhoBTB1 and then restored METTL7B expression by transfection. This re-expression of METTL7B was able to significantly rescue Golgi integrity (Fig. 3e, f). Intriguingly, expression of METTL7A also promoted recue of normal Golgi morphology (Fig. 3e, f). We conclude that the effects of RhoBTB1 depletion on Golgi integrity are mediated through the consequent reduced expression of METTL7B, but that METTL7A overexpression can substitute for this activity.

**Fig. 4:**
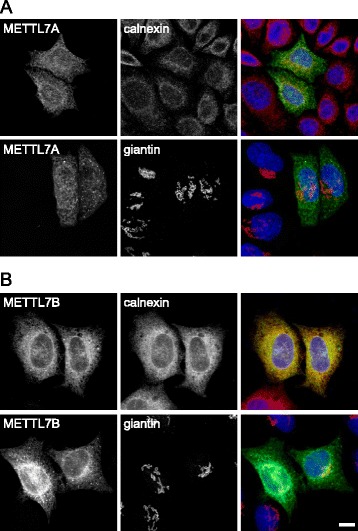
METTL7 proteins are localized to the endoplasmic reticulum. **a** HeLa cells were transfected with myc-tagged METTL7A (*green*) and fixed and stained for the ER marker calnexin (*red*) or the Golgi marker giantin (*red*). METTL7A showed no significant localization to the Golgi, but instead was present in the ER and also to small punctate structures. **b** The experiment was repeated with myc-tagged METTL7B. METTL7B also localized to the ER and small puncta. Bar = 10 μm

METTL7B has been reported as being localized to the Golgi [20], whereas METTL7A (a.k.a. AAM-B) has previously been shown to localize to the endoplasmic reticulum (ER) and to lipid droplets [22]. It was unclear why both isoforms should affect Golgi morphology, given their different locations. We examined the cellular localization of both proteins. In contrast to the previous report [20], we found that both METTL7s localized to the ER in HeLa cells, with no apparent Golgi localization (Fig. 4a, b). We conclude that the relevant target(s) for METTL7 action is likely to be an ER resident protein, or a protein that actively shuttles between the two compartments.

### RhoBTB1 is expression is downregulated in breast cancer cell lines

**Fig. 5:**
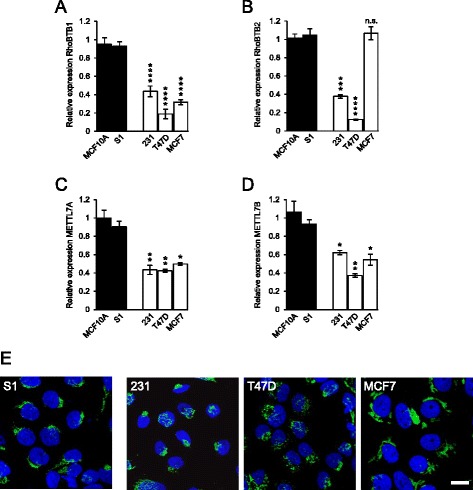
RhoBTB1 is expression is downregulated in breast cancer cell lines. **a-d** The expression of RhoBTB1, RhoBTB2, METTL7A and METTL7B was measured by RT-PCR in three well-characterized breast cancer cell lines: MDA-MB-231, T47D and MCF7. Expression was compared with two well-characterized normal mammary epithelial cell lines: MCF-10A and HMT-S1. Data are means ± SEM (*n* = 3). Data were normalized to HeLa cell samples. Comparisons are to the MCF10A control; *P <0.05; **P <0.01, ***P <0.001, ****P <0.0001. All three breast cancer lines showed a markedly reduced RhoBTB1 expression, as well as reduction in expression of METTL7A and METTL7B. T47D cells had the lowest levels of both RhoBTB1 and RhoBTB2. **e** the breast cancer cell lines were fixed and stained for giantin (*green*) and Golgi morphology compared to the normal mammary epithelial HMT-S1 line. T47D cells showed marked fragmentation of the Golgi. Bar = 10 μm

**Fig. 6:**
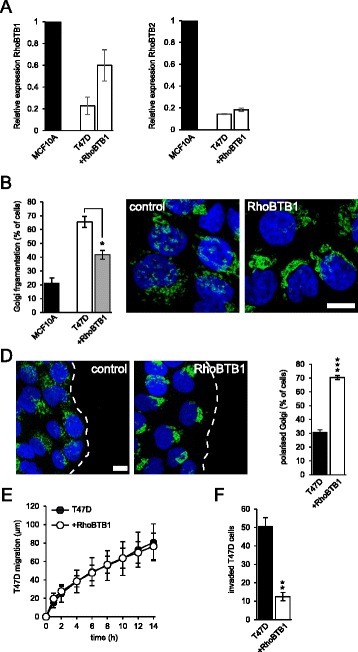
Restoration of RhoBTB1 expression restores normal Golgi morphology to T47D cells and inhibits invasion. **a** T47D cells were transduced with a lentiviral RhoBTB1 construct to restore RhoBTB1 expression. Expression of RhoBTB1 and RhoBTB2 was measured by RT-PCR. Transduction with RhoBTB1 restored expression to near normal levels, but did not affect expression of RhoBTB2. Data are means ± SEM (*n* = 2). **b** Cells were fixed and stained for the Golgi marker giantin. Restoration of RhoBTB1 expression caused a significant reduction in Golgi fragmentation. Data are means ± SEM (*n* = 3). **c** shows representative images of control T47D cells, and the T47D line expressing RhoBTB1. Golgi are stained in green; nuclei in *blue*. Bar = 10 μm. **d** T47D cells were grown to confluence and cell migration was initiated by scratching the coverslip to remove a strip of cells. Cells were fixed at 1 h and stained for Golgi (giantin, *green*). The dashed line indicates the cell front. Quantification of Golgi polarization showed that T47D cells had little ability to polarize (25% polarization corresponds to random orientation in this assay); whereas restoration of RhoBTB1 supported robust polarization. Data are means ± SEM (*n* = 3). **e** Migration was measured in the same assay over 14 h. Data are means ± SEM (*n* = 3). Restoration of RhoBTB1 expression had no effect on T47D cell migration in 2D. **f** T47D invasion through 3D extracellular matrix was measured in Matrigel invasion chambers over 24 h. Restoration of RhoBTB1 expression markedly reduced invasive capacity. Data are means ± SEM (*n* = 3). *P <0.05; **P <0.01, ***P <0.001, ****P <0.0001

Many studies have reported downregulation of RhoBTB2 expression in breast cancer, most commonly due to methylation of the RhoBTB2 promoter [7, 10, 11, 23, 24]. We examined the relative expression of RhoBTB1 and RhoBTB2 in a selection of human breast cancer cell lines (MDA-MB-231, T47D, MCF7) in comparison to two well-characterized normal human mammary epithelial cell lines – MCF10A [25] and HMT-3522 S1 [26]. All three breast cancer cell lines had reduced RhoBTB1 expression (Fig. 5a). T47D and MDA-MB-231 cells also showed reduced expression of RhoBTB2, whereas expression was normal in MCF7 cells (Fig. 5b). We examined the expression of METTL7A and METTL7B in the same cell lines. Interestingly, both isoforms showed reduced expression in the breast cancer cell lines, with T47D cells having the lowest overall expression of METTL7 isoforms (Fig. 5c, d). T47D cells have previously been shown to undergone loss of RhoBTB2 expression [7]. They also had the lowest expression of RhoBTB1 (Fig. 5a). Intriguingly, whereas the normal mammary cell lines had a compact Golgi ribbon, T47D cells showed extreme fragmentation of the Golgi (Figs. 5e, 6b), raising the possibility of a functional link between loss of RhoBTB1 expression and Golgi morphology in this breast cancer line.

### Restoration of RhoBTB1 expression restores normal Golgi morphology to T47D cells and inhibits invasion

To explore the effect of loss of RhoBTB1 expression on Golgi integrity in T47D cells, we restored RhoBTB1 expression using lentiviral transduction. The resultant stable cell line had near normal levels of RhoBTB1, but still had dramatically lower levels of RhoBTB2 (Fig. 6a). Restoration of RhoBTB1 expression led to a significant rescue of Golgi integrity (Fig. 6b).

Fragmentation of the Golgi has little effect on the rate of protein secretion in cells [27]. In keeping with this, we saw no apparent change in the rate of trafficking of VSV-G through the secretory pathway in cells depleted of RhoBTB1 (data not shown). One proposed function of the Golgi ribbon structure is to facilitate the cellular localization of this organelle. Polarized epithelial cells orientate their Golgi towards the apical surface of the cell, and this is important for polarized secretion and for maintenance of the apical-basolateral axis [28]. In migrating cells, the Golgi is polarized towards the leading edge of the cell in the majority of cell types and this reinforces directional migration [29]. Dysregulation of cell polarity pathways is an important component in the conversion of cancer cells into a migratory, invasive phenotype. We examined whether restoration of RhoBTB1 expression and normalization of Golgi morphology would affect these processes in T47D breast cancer cells. We used a scratch migration assay to measure Golgi polarization and the directional migration of cells. T47D cells failed to polarize their Golgi effectively in the scratch assay; whereas restoration of RhoBTB1 expression resulted in strong polarization of the Golgi towards the migration front (Fig. 6d). Restoration of RhoBTB1 expression had no effect on T47D cell migration, however, suggesting that the Golgi fragmentation observed in T47D cells does not affect their migration in 2D (Fig. 6e). We next examined the invasive capacity of these cells. A critical early stage in breast cancer progression is breakdown of the highly polarized organization of cells in a mammary duct and their invasion through the basement lamina and into the surrounding stroma. We measured the ability of T47D breast cancer cells to invade through a 3D layer of extracellular matrix that mimics the composition of the basement lamina [30]. Restoration of RhoBTB1 expression dramatically inhibited cell invasion (Fig. 6f). We conclude that loss of RhoBTB1 expression in T47D breast cancer cells contributes to a pro-invasion phenotype. Our hypothesis is that this is a result of reduced METTL7B expression, leading to Golgi fragmentation and a breakdown of normal cell polarity.

## Discussion

RhoBTB proteins are highly unusual members of the RhoGTPase family. Their multidomain structure makes them atypical; however, they are present in a wide range of organisms, including the social amoeba *Dictyostelium discoideum*, where they were first identified [31]. We still know very little about the cellular functions of RhoBTB1 and 2. Most progress has come from investigating the function of their twin BTB domains [6]. In many proteins, the BTB domain functions to recruit the Cul3 ubiquitin ligase [32]. RhoBTB2 can bind Cul3 through its first BTB domain [33, 34]. RhoBTB2 itself is a target of ubiquitylation by this complex, leading to its subsequent degradation by the proteasome [34]. This raises the possibility of a role for the RhoBTB2/Cul3 complex in regulating the turnover of other cellular proteins, although no targets of RhoBTB2/Cul3 mediated ubiquitylation have yet been identified.

BTB domains are also frequently found in transcriptional regulators, where they have a number of roles, including the recruitment of transcriptional co-repressors [35]. Previously, we showed that RhoBTB2 controls the expression of the CXCL14 chemokine in epithelial cells [16]. Here were show that the METTL7 enzymes are targets of transcriptional control by RhoBTB1 and 2. CXCL14 expression requires both RhoBTB1 and 2 [16], whereas the two RhoBTBs had specific roles in regulating the METTL7 enzymes, with RhoBTB2 regulating expression of METTL7A and RhoBTB1 regulating METTL7B. The RhoBTBs dimerize through their BTB domains, and can form both homo- and heterodimers [33]. It seems likely these different dimer pairs have different transcriptional targets. This becomes important when considering the effects of the downregulation of RhoBTB1 and 2 in cancer cells, where loss of one isoform could potentially increase homodimer concentration of the other, in addition to the simpler effects of reducing its own concentration.

We show here that RhoBTB1 regulates Golgi integrity. Interestingly, the third RhoBTB family member, RhoBTB3, has also been shown to regulate Golgi function. RhoBTB3 is only weakly-related to RhoBTB1 and 2, but shares a similar domain organization [6]. RhoBTB3 is a Golgi-localized protein and binds to directly to the secretory pathway regulator Rab9 to mediate trafficking from the endoplasmic reticulum to the Golgi [36]. RhoBTB3 silencing has been reported to lead to enlargement of the Golgi [36] and in a later study to cause Golgi fragmentation[37] The mechanism by which RhoBTB3 affects Golgi integrity has not been reported. We show here that RhoBTB1 controls Golgi integrity through regulation of the expression of METTL7B. The downstream targets of the poorly-characterized METTL7 enzymes are unknown. Both contain an N-terminal transmembrane domain and a C-terminal methyltransferase domain, predicted to lie on the cytoplasmic face. Interestingly, METTL7B undergoes arginine dimethylation [20], raising the possibility of automethylation. Depletion of either METTL7A or B caused Golgi fragmentation (Fig. 3c) and METTL7A overexpression was able to rescue the effects of depletion of METTL7B (Fig. 3e). This suggests that the two proteins have overlapping functions in regulating Golgi integrity. Silencing of RhoBTB2 did not cause significant Golgi fragmentation (Fig. 2b), despite a reduction in METTL7A expression (Fig. 1a). The effects of RhoBTB2 on METTL7A are less pronounced than those of RhoBTB1 on METTL7B, at least in HeLa cells (Fig. 1). It is possible that RhoBTB2 might affect Golgi integrity in other cell types, perhaps where METTL7A is the more dominant isoform.

In vertebrates, the Golgi apparatus is organized into a compact ribbon structure, typically located next to the nucleus [38]. This organization depends on the constant action of the motor protein dynein and on the functions of Golgi structural proteins [29, 38]. Surprisingly, Golgi ribbon structure is not requisite for the functioning of the secretory pathway. In plants, individual Golgi stacks are scattered throughout the cytosol, and in the yeast *Saccharomyces cerevisiae*, the Golgi is composed of dispersed, isolated cisternae [38, 39]. In vertebrate cells, treatments that fragment the Golgi do not affect the rate of protein secretion [40, 41]. Instead, the organized Golgi ribbon structure seems to be important for vertebrate cell polarization. In polarized epithelial cells, the Golgi is orientated towards the apical surface [28]. In most migrating vertebrate cells, the Golgi is orientated towards the leading edge [29, 42]. These observations suggest that a polarized Golgi ribbon may facilitate polarized secretion. In polarized epithelial cells, this would support apical/basolateral polarity, and in migrating cells, it would contribute to directional movement. In keeping with this, recent work supports a role for the Golgi in the polarized delivery of active Cdc42 to the leading edge of migrating cells [43]. Fragmentation of the Golgi has previously been shown to inhibit the migration of HeLa cells [27]. Here we find that the loss of expression of RhoBTB1 in T47D breast cancer cells is linked to fragmentation of the Golgi. This can be rescued by re-expression of RhoBTB1; however, this has no effect on migration of these cells in 2D (Fig. 6e). Instead, we found that re-expression of RhoBTB1 in T47D breast cancer cells strongly inhibited their invasive capacity in 3D (Fig. 6f). The initial stages of breast cancer progression involve loss of apical-basolateral polarity and invasion through the basement lamina. We propose that loss of RhoBTB1 early in breast cancer development promotes loss of normal epithelial polarity through reduced METTL7B expression and Golgi fragmentation. We propose that this then contributes to the switch to an invasive phenotype.

## Conclusions

RhoBTB1 is important for maintaining the integrity of the Golgi, through regulation of the expression of METTL7B. In keeping with this, loss of RhoBTB1 expression leads to fragmentation of the Golgi. Restoration of normal RhoBTB1 levels to T47D breast cancer cells restores Golgi integrity and leads to a dramatic decrease in their invasive capacity.

## Additional file

Additional file 1: Details of antibodies, plasmids and oligonucleotides used in the study. (DOCX 13 kb)

## Abbreviations

BTB: Broad-complex, tramtrack and bric a brac
ER: Endoplasmic reticulum
HNSC: Head and neck squamous cell
METTL: Methyltransferase like
RT-PCR: Real-time PCR.
