# Supplementary material for: The tumor suppressor RhoBTB1 controls Golgi integrity and breast cancer cell invasion through METTL7B
Source: BMC Cancer. 2017 Feb 20;17:145. doi: 10.1186/s12885-017-3138-3 (PMC5319017; doi:10.1186/s12885-017-3138-3)
Supplement: Additional file 1: — Details of antibodies, plasmids and oligonucleotides used in the study. (DOCX 13 kb) [file 12885_2017_3138_MOESM1_ESM.docx]

**Additional file 1**

**Table 1**

**Antibodies and fluorescent probes used in the study**

| **Antibody/probe** | **Type** | **Source** | **Cat. #** | **Use** |
| --- | --- | --- | --- | --- |
| giantin | rabbit polyclonal | Covance | PRB-114P | Imaging |
| calnexin | rabbit polyclonal | Dr Jon Lane | - | Imaging |
| myc epitope | mouse monoclonal  (clone 9E10) | Cancer Research UK | - | Imaging |
| Alexa-594 donkey anti-mouse IgG | donkey polyclonal | Life Technologies | 21203 | Imaging |
| Alexa-488 donkey anti-rabbit IgG | donkey polyclonal | Life Technologies | 21207 | Imaging |

**siRNAs used in the study**

| **Name** | **Target** | **Sequence** |
| --- | --- | --- |
| lamin | lamin A/C | GGUGGUGACGAUCUGGGCUTT |
| RhoBTB1-A | RhoBTB1 | GCAGUUGUCUCCUAACUUGTT |
| RhoBTB1-B | RhoBTB1 | UGCAAGCAGUAUUGGAUUATT |
| RhoBTB2-A | RhoBTB2 | UGACAUGGAUUAUGAAAGGUU |
| RhoBTB2-B | RhoBTB2 | GACCGUCGCUUUGCUUAUGUU |
| M7A-A | METTL7A | GUGUUCGACUUGGAAUUACTT |
| M7A-B | METTL7A | UAGUGUGAGCUGGCAGUUATT |
| M7B-A | METTL7B | AUUGUCAAGUAGGAGACAATT |
| M7B-B | METTL7B | GACAUUCAUGUACCACCUA |

**Q-PCR primers used in the study**

| **Name** | **Target** | **Sequence** |
| --- | --- | --- |
| RPOL-f | RNA polymerase II | GCACCACGTCCAATGACAT |
| RPOL-r | RNA polymerase II | GTGCGGCTGCTTCCATAA |
| RhoBTB1-f | RhoBTB1 | CCCTTCCTACCTCCAAAAGC |
| RhoBTB2-r | RhoBTB1 | CTCACAGGCTCCTTCACTCC |
| RhoBTB2-f | RhoBTB2 | TGGGTAAGACCAGGCTCATC |
| RhoBTB2-r | RhoBTB2 | GTCTCCAAAGGTGTCCCAGA |
| METTL7A-f | METTL7A | GAACTTTCTGGGCTTGTGGA |
| METTL7A-r | METTL7A | CCCAGGTGGGTAGAACTTGA |
| METTL7B-f | METTL7B | TGCTCTTTTTCTGGGAGCAT |
| METTL7B-r | METTL7B | GCTGTCGTTCCATTTGGATT |

**Plasmids used in the study**

| **Name** | **Vector** | **Insert** |
| --- | --- | --- |
| myc-METTL7A | pcDNA3 | human METTL7A with C-terminal myc-tag |
| myc-METTL7B | pcDNA3 | human METTL7B with C-terminal myc-tag |
| mCherry-RhoBTB1 | pHR’SIN-cPPT-SEW | Human RhoBTB1 with an N-terminal mCherry tag cloned into a lentiviral vector |
